# Acute kidney injury risk in orthopaedic trauma patients pre and post surgery using a biomarker algorithm and clinical risk score

**DOI:** 10.1038/s41598-020-76929-y

**Published:** 2020-11-17

**Authors:** Mary Jo Kurth, William T. McBride, Gavin McLean, Joanne Watt, Anna Domanska, John V. Lamont, Daniel Maguire, Peter Fitzgerald, Mark W. Ruddock

**Affiliations:** 1 Randox Laboratories Ltd, 55 Diamond Road, Crumlin, County Antrim BT29 4QY Northern Ireland, UK; 2 Department of Cardiac Anaesthesia, Royal Victoria Hospital, Belfast Health and Social Care Trust, 274 Grosvenor Road, Belfast, BT12 6BA Northern Ireland, UK; 3 Trauma and Orthopaedics, Craigavon Area Hospital, 68 Lurgan Road, Portadown, Craigavon, BT63 5QQ Northern Ireland, UK

**Keywords:** Biomarkers, Medical research, Nephrology, Pathogenesis, Risk factors

## Abstract

Acute kidney injury (AKI) after major trauma is associated with increased mortality. The aim of this study was to assess if measurement of blood biomarkers in combination with clinical characteristics could be used to develop a tool to assist clinicians in identifying which orthopaedic trauma patients are at risk of AKI. This is a prospective study of 237 orthopaedic trauma patients who were consecutively scheduled for open reduction and internal fixation of their fracture between May 2012 and August 2013. Clinical characteristics were recorded, and 28 biomarkers were analysed in patient blood samples. Post operatively a combination of H-FABP, sTNFR1 and MK had the highest predictive ability to identify patients at risk of developing AKI (AUROC 0.885). Three clinical characteristics; age, dementia and hypertension were identified in the orthopaedic trauma patients as potential risks for the development of AKI. Combining biomarker data with clinical characteristics allowed us to develop a proactive AKI clinical tool, which grouped patients into four risk categories that were associated with a clinical management regime that impacted patient care, management, length of hospital stay, and efficient use of hospital resources.

## Introduction

Acute kidney injury (AKI) after major trauma is associated with increased mortality. In a recent meta-analysis, from 17 articles describing AKI outcomes in 24,246 trauma patients, the pooled incidence of AKI was 20.4%^1^. The Kidney Disease: Improving Global Outcomes (KDIGO) criteria have reported that the incidence of AKI is 7–11% after orthopaedic surgery^2,3^. Orthopaedic trauma patients in general already have longer hospital stay, higher post-operative morbidity and mortality, and reduced quality of life^4^.

The most recent criteria developed for the diagnosis of AKI is the 2012 KDIGO^5^, which combines Risk/Injury/Failure/Loss/End-stage (RIFLE) and Acute Kidney Injury Network (AKIN) and relies on changes in serum creatinine (sCr) levels and urine output. Urine output may be compromised as a result of surgery and if used as an indicator of AKI could misclassify patients^6^. Creatinine measured in serum or plasma is the most commonly used biomarker to determine estimated glomerular filtration rate (eGFR). However, an increase in creatinine levels after renal insult is often delayed and a change in plasma creatinine levels may not be evident until two to three days after the initial insult^7^. Work in animals and humans has shown that although AKI due to ischemia can be prevented and treated, preventative therapy must be started very early after the renal injury^8^.

Single biomarkers are unlikely to predict or diagnose AKI due to the heterogeneity involved in the pathogenesis of AKI. Identification of biomarkers for AKI, have focussed on cardiac surgery populations^9–14^. Indeed, a recent publication identified a biomarker combination (heart-type fatty acid binding protein (H-FABP), midkine (MK) and soluble tumour necrosis factor (sTNFR) 1 or 2) that predicted AKI both pre and post cardiac surgery^11^. Clinical risk factors for AKI have been reported to include age, male gender, pre-existing chronic kidney disease (CKD), diabetes, heart failure and surgery^15–17^. Interestingly, clinical risk factors, pre and post cardiac surgery, identified in a recent publication, when combined with biomarkers could predict the risk of AKI^11^. However, biomarker combinations together with clinical risk factors have not been identified for orthopaedic trauma patients.

Therefore, we considered (1) could measurement of blood biomarkers pre and post surgery be used to stratify risk of AKI in orthopaedic trauma patients? and (2) could biomarker data combined with clinical characteristics be used to develop a tool to assist clinicians in identifying orthopaedic trauma patients at risk of AKI and guide patient management?

## Methods

### Study population

**Figure 1:**
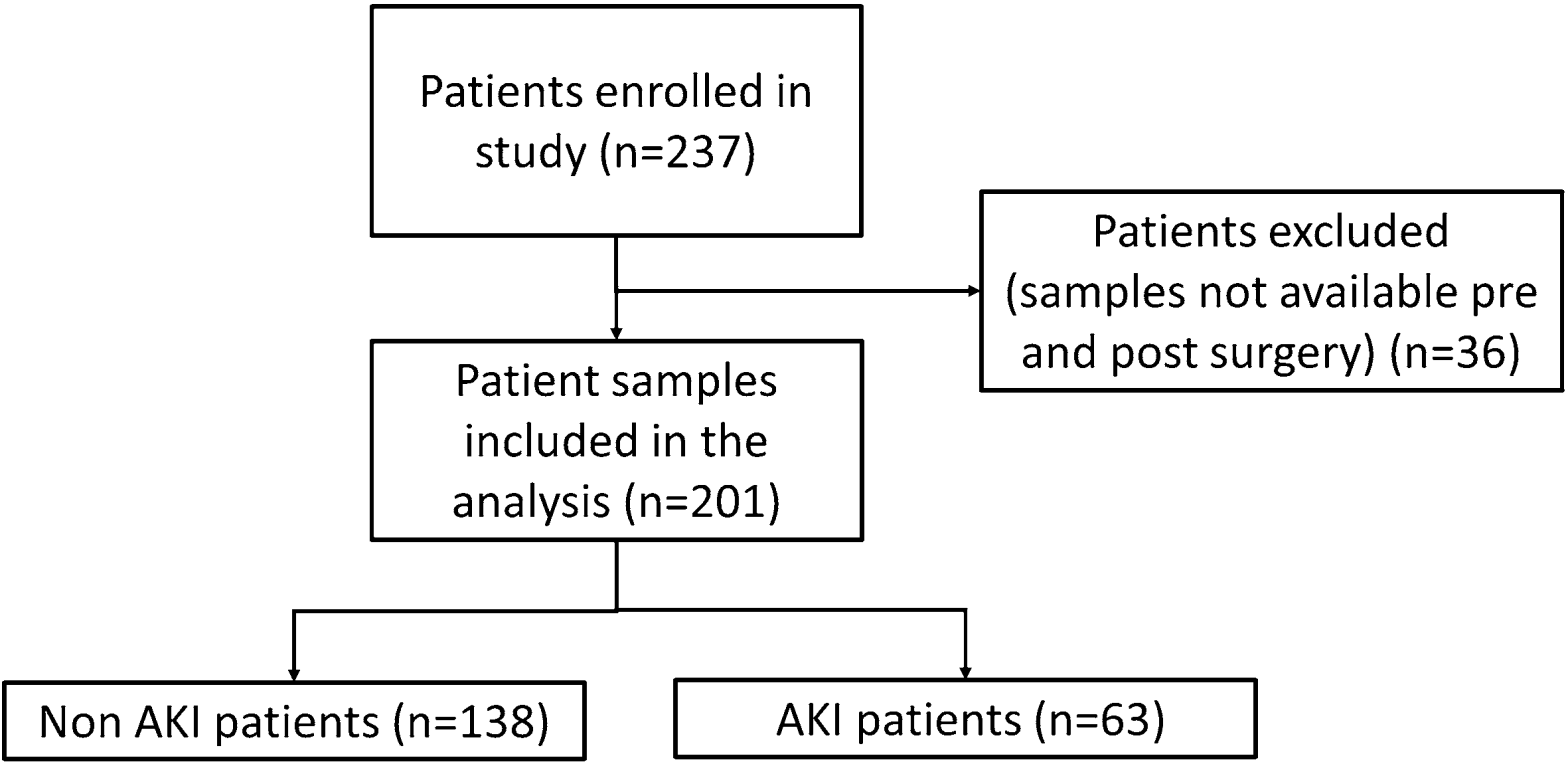
Trial flow diagram. *AKI* acute kidney injury.

This prospective study of 237 patients was performed within the Fracture Unit of the Royal Victoria Hospital, Belfast, UK between May 2012 and August 2013. The study complied with the Declaration of Helsinki, was approved by the Office for Research Ethics Committee Northern Ireland, the Royal Victoria Hospital Research Office Research Governance Committee and written informed consent was obtained from all participating patients. Orthopaedic trauma patients who were consecutively scheduled for open reduction and internal fixation (ORIF) of their fracture, were recruited into the study. Patients were excluded if they were < 18 years of age, had preoperative or pre-trauma dialysis-dependent renal failure or had a history of significant renal disease prior to recruitment. Of the n = 237 patients recruited to the study, pre and post operative samples were available for 201/237 (84.8%) patients. Patient samples were not available for 36/237 (15.2%) and these patients were excluded from the study (Fig. 1).

### Clinical data collection

Clinical data was recorded for each patient from medical records that included baseline demographic characteristics, comorbidities and current medications.

### Sampling and laboratory methods

Patient blood samples (10 ml) were collected preoperatively and on day 1 post operatively. Patient blood samples were centrifuged, and serum and plasma were aliquoted within 30 min of collection and stored at − 80 °C.

Patient blood samples were analysed in duplicate by Randox Clinical Laboratory Services (RCLS) (Antrim, UK) using cytokine arrays (Randox Laboratories Ltd, Crumlin, UK), ELISAs or IT assays, as described previously^11^. Biomarker values below the limit of detection (LOD) were recorded as 90% of LOD. A total of 27 biomarkers (IL-2 (LOD 4.9 pg/ml), IL-4 (LOD 3.5 pg/ml), IL-6 (LOD 0.4 pg/ml), IL-8 (LOD 2.3 pg/ml), VEGF (LOD 10.8 pg/ml), IFNγ (LOD 2.1 pg/ml), TNFα (LOD 3.7 pg/ml), IL-1α (LOD 0.9 pg/ml), MCP-1 (LOD 25.5 pg/ml), EGF (LOD 2.5 pg/ml), IL-10 (LOD 1.1 pg/ml), IL-1β (LOD 1.3 pg/ml), IL-1Rα (LOD 16.83 pg/ml), PDGF-BB (LOD 16.16 pg/ml), IP-10 (LOD 7.81 pg/ml), IL12-p40 (LOD 7.81 pg/ml), sIL-2A (LOD 0.12 ng/ml), sIL-6R (LOD 0.62 ng/ml), sTNFR1 (LOD 0.09 ng/ml), sTNFR2 (LOD 0.2 ng/ml), MMP-9 (LOD 3.03 ng/ml), CRP (LOD 0.67 mg/l), D-Dimer (LOD 2.1 ng/ml), NSE (LOD 0.26 ng/ml), NGAL (LOD 17.8 ng/ml), MK (LOD 8.0 pg/ml) and H-FABP (LOD 2.94 ng/ml) were measured by RCLS. Serum creatinine (LOD 5 μm/L) was measured in the Kelvin Laboratory, Belfast Royal Victoria Hospital, Belfast.

### Outcome definition

Patients did not have a baseline eGFR measurement prior to trauma but were assumed to have a normal renal function with a baseline eGFR of at least 60 ml/min/1.73m^2^^18–20^. A value of < 45 ml/min/1.73m^2^ was used to define a patient as AKI positive on any of the recorded pre and post operative sampling days, in accordance with the RIFLE classification^21^; any patient with an eGFR result at any time (day 0, 1, 2, and 5) > 25% of 60 ml/min/1.73m^2^ (45 ml/min/1.73m^2^) were determined to have AKI.

### Statistical analysis

Statistical analyses were performed using R^22^. Wilcoxon rank sum test was used to identify differentially expressed biomarkers. Biomarkers with a p < 0.05 were considered significant. The ability of the biomarkers to predict AKI was further investigated using logistic regression (Lasso regression). For each biomarker and biomarker combination, areas under the receiver operator characteristic (AUROC), sensitivity, specificity, positive predictive value (PPV) and negative predictive value (NPV) were generated pre and post operatively to identify models that differentiated between the two diagnostic groups (non AKI vs. AKI).

## Results

**Figure 2:**
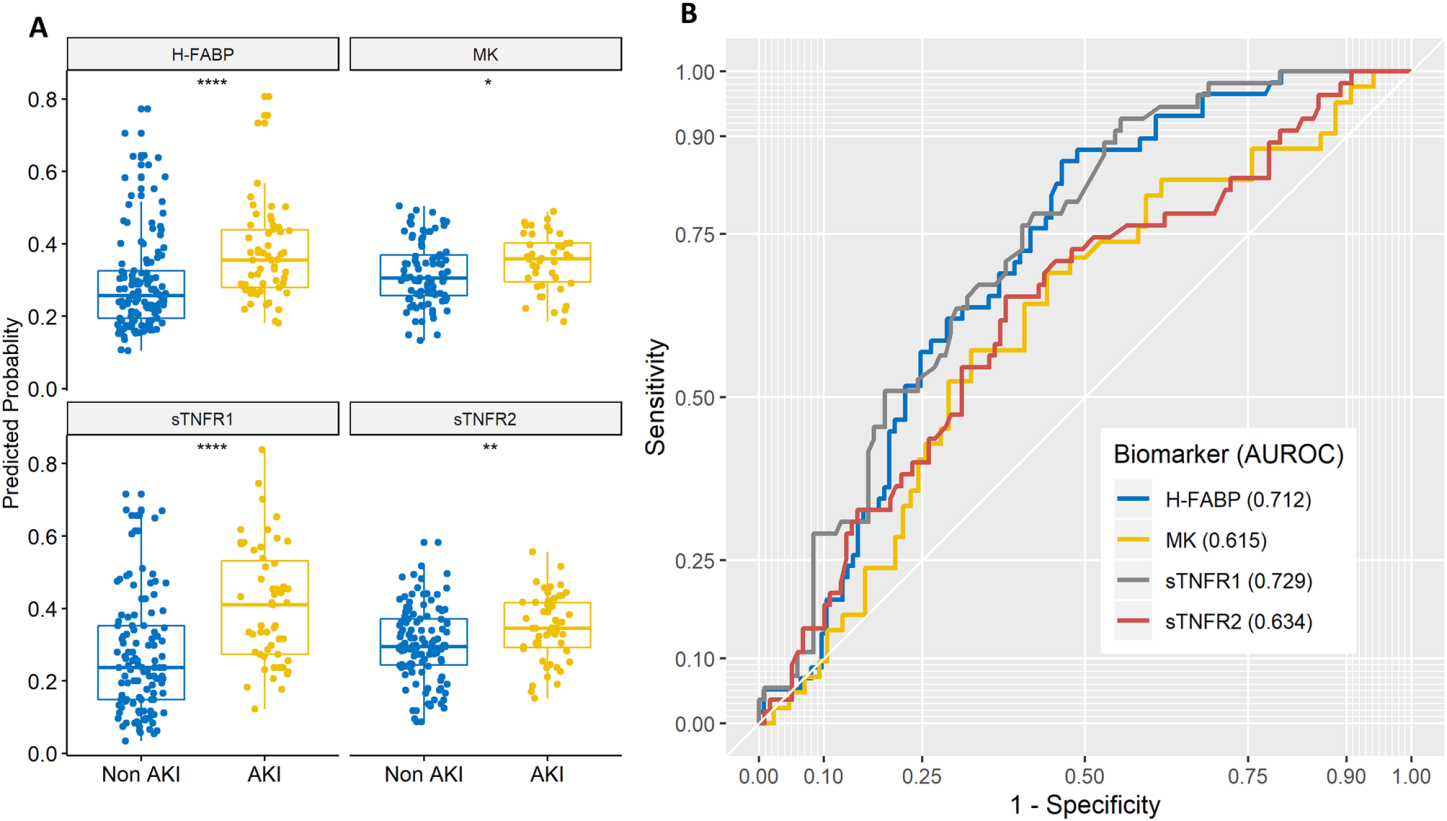
(**A**) Pre surgery serum biomarker predicted probabilities for AKI development post operatively. H-FABP, MK, sTNFR1 and sTNFR2 predicted probabilities for preoperative serum levels. Wilcoxon rank sum statistical significance is indicated by *p ≤ 0.05, **p ≤ 0.01, ****p ≤ 0.0001. *AKI* acute kidney injury, *H-FABP* heart-type fatty acid-binding protein, *MK* midkine, *sTNFR* soluble tumour necrosis factor receptor. (**B**) Receiver operator characteristics for pre surgery serum biomarkers. H-FABP (AUROC 0.712), MK (AUROC 0.615), sTNFR1 (AUROC 0.729) and sTNFR2 (AUROC 0.634). *AUROC* area under the receiver operator characteristic, *H-FABP* heart-type fatty acid-binding protein, *MK* midkine, *sTNFR* soluble tumour necrosis factor receptor.

**Figure 3:**
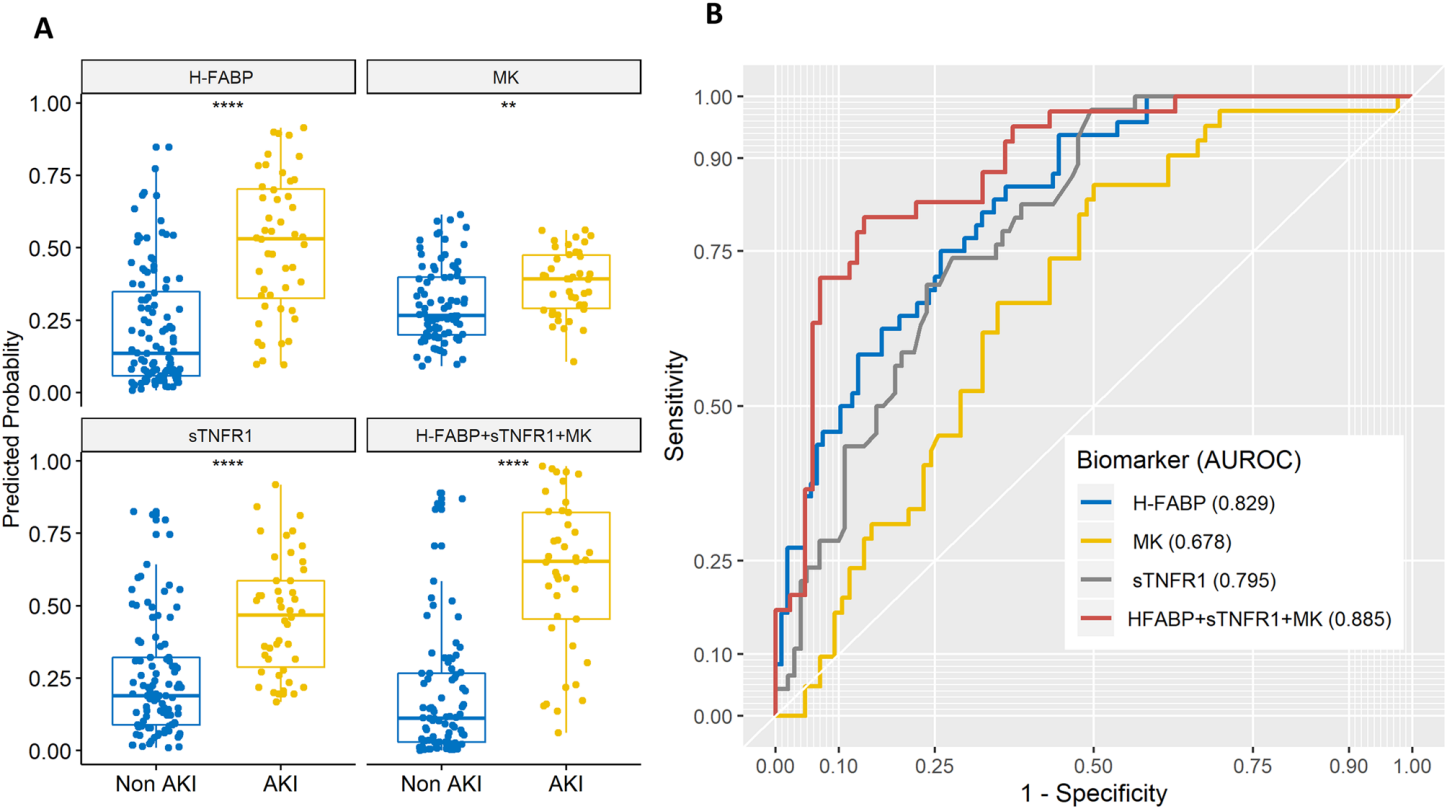
(**A**) Post operative serum biomarker model predicted probabilities for non AKI and AKI patients. Predicted probabilities for post surgery serum levels for H-FABP, MK and sTNFR1 individually and combined. Wilcoxon rank sum statistical significance is indicated by: **p <  = 0.01, ****p <  = 0.0001. *AKI* acute kidney injury, *H-FABP* heart-type fatty acid-binding protein, *MK* midkine, *sTNFR* soluble tumour necrosis factor receptor. (**B**) Receiver operator characteristics for post surgery serum biomarkers and model. H-FABP (AUROC 0.829), MK (AUROC 0.678), sTNFR1 (AUROC 0.795) and model H-FABP + MK + sTNFR1 (AUROC 0.885). *AUROC* area under the receiver operator characteristic, *H-FABP* heart-type fatty acid-binding protein, *MK* midkine, *sTNFR* soluble tumour necrosis factor receptor.

Clinical characteristics for patients involved in the study are presented in Table 1. Of the 28 biomarkers that were investigated, sTNFR1 and H-FABP had the highest AUROC pre surgery to stratify risk of AKI in orthopaedic trauma patients (Table 2) (sTNFR1 sensitivity 76.4%; specificity 59.7%; AUROC 0.729 (CI 0.654–0.804); H-FABP sensitivity 62.1%; specificity 71.2%; AUROC 0.712 (CI 0.637–0.786) (Fig. 2A,B)). LASSO regression identified a combination of 3 biomarkers post operatively to stratify risk of AKI, namely H-FABP, sTNFR1 and MK (Table 2) (H-FABP, sTNFR1 and MK combined sensitivity 80.5%; specificity 86.0%; AUROC 0.885 (CI 0.825—0.944) (Fig. 3A,B)).

**Table 1:** Summary of clinical characteristics of the study patients.

	Non AKI (n = 138)	AKI (n = 63)	p value
**Patient characteristics**			
Age (years)	78.7 ± 10.9	85.5 ± 6.1	0.000
Gender (female)	109/138 (79.0%)	42/63 (55.7%)	0.089
**Comorbidities**			
Hypertension	38/138 (27.5%)	27/63 (42.9%)	0.046
Diabetes	11/138 (8.0%)	4/63 (6.3%)	0.907
Dementia	15/138 (10.9%)	16/63 (25.4%)	0.015
**Pre surgery medications**			
Hypertensive medications	46/116 (40.5%)	29/51 (56.9%)	0.074
**Intraoperative conditions**			
Phenylephrine	19/115 (16.5%)	14/52 (26.9%)	0.176
Packed red blood cells	6/115 (5.2%)	2/52 (3.8%)	1.000
Fresh frozen plasma	0/115 (0.0%)	1/52 (1.9%)	0.683
Platelet bags	4/115 (3.5%)	2/52 (3.8%)	1.000
**Operative method**			
Hemiarthroplasty	30/138 (43.5%)	36/63 (57.1%)	0.100
Intramedullary nailing	14/138 (10.1%)	1/63 (1.6%)	0.064
Sliding hip screw	54/138 (39.1%)	26/63 (41.3%)	0.895
Total hip replacement	10/138 (7.2%)	0/63 (0.0%)	0.065
**Post operative conditions**			
Packed red blood cells	34/115 (29.6%)	16/52 (30.8%)	1.000
Fresh frozen plasma	0/115 (0.0%)	1/52 (1.9%)	0.683
**Other**			
Hospital stay (days)	9.8 ± 7.9	12.0 ± 8.3	0.020
Operation time (minutes)	53.8 ± 19.1	52.4 ± 18.4	0.636
Time between presentation and surgery (days)	2.1 ± 1.5	2.5 ± 2.0	0.138

Data presented as mean ± standard deviation or number/total (%).

*AKI* acute kidney injury.

**Table 2:** Serum biomarkers for predicting AKI pre and post surgery.

	Anytime						
	Biomarkers (n)	AUROC	CI	Sensitivity (%)	Specificity (%)	PPV (%)	NPV (%)
Pre operative	MK (128)	0.615	0.513–0.718	57.1	67.4	46.2	76.3
	sTNFR2 (174)	0.634	0.546–0.723	65.5	62.2	44.4	79.6
	H-FABP (183)	0.712	0.637–0.786	62.1	71.2	50.0	80.2
	sTNFR1 (174)	0.729	0.654–0.804	76.4	59.7	46.7	84.5
Post operative	MK (128)	0.678	0.585–0.772	66.7	65.1	48.3	80.0
	sTNFR2 (147)	0.734	0.648–0.821	67.4	71.3	51.7	82.8
	sTNFR1 (147)	0.795	0.724–0.866	73.9	72.3	54.8	85.9
	H-FABP (156)	0.829	0.764–0.893	75.0	74.1	56.3	87.0
	H-FABP + sTNFR2 (147)	0.866	0.809–0.924	80.4	81.2	66.1	90.1
	H-FABP + sTNFR2 + MK (127)	0.870	0.809–0.932	78.0	84.9	71.1	89.0
	H-FABP + sTNFR1 (147)	0.881	0.825–0.937	78.3	87.1	73.5	89.8
	H-FABP + sTNFR1 + MK (127)	0.885	0.825–0.944	80.5	86.0	73.3	90.2

AUROC, CI, sensitivity, specificity, PPV and NPV for serum biomarkers for predicting AKI pre and post surgery.

*n* number, *AKI* acute kidney injury, *AUROC* area under the receiver operator characteristic, *CI* confidence interval, *MK* midkine, *NPV* negative predictive value, *PPV* positive predictive value, *sTNFR* soluble tumour necrosis factor receptor, *H-FABP* heart-type fatty acid-binding protein.

To develop a tool to assist clinicians in identifying orthopaedic trauma patients at risk of AKI and guide patient management, the clinical characteristics between non AKI and AKI patients were investigated. We used biomarker combinations to determine a biomarker risk score (BRS) pre and post surgery (based on AUROC determined by LASSO regression) that could be used to identify patients at risk of AKI. Establishing a biomarker set point (cut-off) pre and post surgery categorised patients either positive or negative for AKI i.e. if a post surgery patient had a BRS above the set point they would be predicted to be positive for AKI (Table 3).

**Table 3:** Post surgery patient score calculation and BRS determination.

BRS	Patient score*
Negative	< − 1.05
Positive	≥ − 1.05

The patient score equation was derived from logistic regression. The cut-off (closest top left) of − 1.05 was determined using the following equation:

closest top left = min((1 − sensitivities)^2^ + (1 − specificities)^2^).

If patient score < − 1.05 then BRS is negative, if patient score ≥ − 1.05 then BRS is positive.

*BRS* biomarker risk score, *H-FABP* heart-type fatty acid-binding protein, *sTNFR* soluble tumour necrosis factor receptor, *MK* midkine, *min* minimum.

*Patient Score = − 8.185 + 2.037*ln(H-FABP) + 2.373*ln(sTNFR1) + 0.056*ln(MK).

Three clinical characteristics were identified for patients at potential risk of AKI pre and post operatively (Table 4). Each clinical characteristic was given a score 0 or 1 (0 = no risk, 1 = risk). Each clinical characteristic was then added to give a cumulative risk score (CRS). For example, pre surgery patients who score ≥ 1 e.g. an ≥ 80-year-old patient with dementia and hypertension would have a cumulative CRS of 3 and would therefore be categorized high risk for AKI. The cut-off for age was based on significance, where patients > 80 years were at greater risk of AKI, based on our patient cohort.

**Table 4:** Clinical risk factors.

Clinical factor	Level	Clinical factor score
Age	< 80	0
	≥ 80	1
Dementia	No	0
	Yes	1
Hypertension	No	0
	Yes	1

If total clinical factor score = 0 then CRS is low, if total clinical factor score ≥ 1 then CRS is high.

*CRS* clinical risk score.

To translate the results of the BRS and CRS into a proactive clinical AKI tool, the BRS and CRS were combined. Combining BRS with CRS either pre or post surgery identified 4 risk categories for patient management (Table 5). Categories 1 and 2 = low risk; Categories 3 and 4 = high risk. Two worked examples for a non AKI and AKI patient, are shown in the Supplementary Notes S1–S3 and Supplementary Tables S1–S6. The distribution of non AKI and AKI within the patient cohort, for each risk category, is shown in Supplementary Note S4.

**Table 5:** Clinical management of patients using a combination of BRS and CRS either pre or post surgery.

Category	BRS	CRS	Clinical management
1	Negative	Low	Routine management
2	Negative	High	Assign to low risk management
3	Positive	Low	Assign to higher risk management
4	Positive	High	Assign to highest risk management

Combining BRS and CRS assigns a patient to a risk category.

*BRS* biomarker risk score, *CRS* clinical risk score.

## Discussion

Hip fracture is the most common serious injury reported in the elderly resulting in long hospital stays, high post-operative morbidity and mortality, and reduced quality of life^4^. Furthermore, AKI after trauma, such as hip fracture is associated with a poor prognosis.

Diagnosis of AKI using sCr and urine output can often result in misdiagnosis. The aim of this study was to further investigate if blood biomarkers and clinical risk factors could be used to identify AKI risk in orthopaedic trauma patients pre and post ORIF surgery in a similar fashion to those identified in patients undergoing cardiac surgery^11^. Interestingly, the same blood biomarkers, H-FABP, Midkine, sTNFR1 or sTNFR2, that predicted AKI in pre and post cardiac surgery patients also identified AKI risk in orthopaedic trauma patients undergoing ORIF surgery. The biomarkers combined with clinical characteristics (age, dementia and hypertension) identified from the study, delivered a proactive clinical AKI tool that could assist clinicians with patient management.

A total of 28 blood biomarkers were investigated. However only two biomarkers, sTNFR1 or H-FABP, were identified as predictive of AKI pre surgery and a combination of three biomarkers, sTNFR1, H-FABP and MK, were predictive for AKI post surgery. Remarkably these biomarkers represent three main pathological processes of AKI. Mechanisms contributing to AKI include (1) perioperative episodes of under perfusion, followed by (2) ischemia reperfusion injury during restoration of normal blood pressure. Inflammatory mediators (3) contribute to and augment the renal injurious effects of this twofold process. Accordingly, an additional separate inflammatory insult arising from other perioperative factors such as coagulation disturbance (which is an important proinflammatory mechanism), can augment the renal injurious effect of hypotension and ischemia reperfusion. Biomarkers have been associated with identification of underlying processes of hypotension (VEFG and H-FABP), IRI (MK) and inflammation (sTNFR1 and 2), which as anti-inflammatory biomarkers are taken as surrogates for the underlying proinflammatory response which drives them^11^.

Elevated sTNFR1 levels have been identified in many clinical conditions e.g. kidney disease^23^, neuropathy, cardiovascular disease and diabetes^24^, and circulating levels of sTNFR1 have been shown to be an independent predictor of CKD progression in elderly patients^25^. Tumour necrosis factor alpha (TNFα) and TNFR2 are almost undetectable in the kidneys of healthy subjects unlike TNFR1 which is expressed within the trans-golgi network of the glomerular endothelium^26^. An increase in the level of sTNFRs in CKD patients has been implicated in declining eGFR^27–29^. Moreover, TNFα acting through TNFR1 has a damaging effect on renal endothelial cells^30^, possibly through iNOS, which would generate intratubular toxic levels of NO, as demonstrated by increased urinary nitrate levels in a porcine model of ischaemia reperfusion-mediated AKI^31^. The elevated anti-inflammatory sTNFR1 response in blood may be driven by an underlying proinflammatory response which includes TNFα^31^. Since monomeric TNFα is much smaller than sTNFR1 and 2, it is more readily filtered by the glomerulus. Accordingly, TNFα is able to cause glomerular injury once it escapes from the moderating biological effect of sTNFR1 or 2. This is consistent with orthopaedic trauma patients who develop AKI having elevated levels of sTNFR1 when compared to non AKI patients.

H-FABP was also predictive of AKI pre operatively, and in combination with sTNFR1 and MK, post operatively. H-FABP, associated with cardiac injury, is released into the bloodstream 30 min after an ischaemic event and peaks at 6 h before returning to normal levels after 24 h^32^. H-FABP has been reported to predict AKI pre and post cardiac surgery^7,11,33^ however, this is the first time that H-FABP has been demonstrated to predict AKI in patients pre and post ORIF surgery.

H-FABP is predominantly expressed in the heart but also at lower levels in skeletal muscle, kidney, stomach, brain and testis^34,35^. The levels of H-FABP in skeletal muscle have been shown to be almost half that found in the heart. Moreover, kidney H-FABP levels are almost two-thirds that found in skeletal muscle^36^. While it is known that H-FABP levels increase in the blood, this may arise from skeletal muscle or renal sources, but it is more likely to be from the heart, which is the largest reservoir of H-FABP in the body. In elderly patients, acute coronary insufficiency is common and would be reflected in elevated H-FABP. Any transient hypoperfusion, which such an event would provoke, could result in a significantly heightened risk of AKI. This is the most likely reason why H-FABP was predictive in this orthopaedic trauma patient cohort.

In addition to sTNFR1 and H-FABP, MK was also identified in the biomarker combination to predict AKI post operatively. The pathophysiological roles of MK are diverse, ranging from AKI to progression of CKD, accompanied by hypertension, renal ischaemia and diabetic nephropathy^37,38^. After ischaemic reperfusion MK is immediately induced in the proximal tubules, leading to the upregulation of macrophage inflammatory protein-2 for neutrophils and monocyte chemotactic protein-1 for macrophages^38^. Eventually, infiltrated inflammatory cells cause severe tubulointerstitial injury. Silencing renal MK expression with anti-sense oligos prevents kidney damage and increases osteogenic activity^39^. Midkine is also involved in chondrogenesis and fracture healing^39^. Interestingly, MK-deficient mice have been shown to display increased bone formation rate and volume^39^. This is the first study, to our knowledge which has identified MK as a biomarker for stratifying patients at risk of AKI following orthopaedic trauma and ORIF surgery.

**Figure 4:**
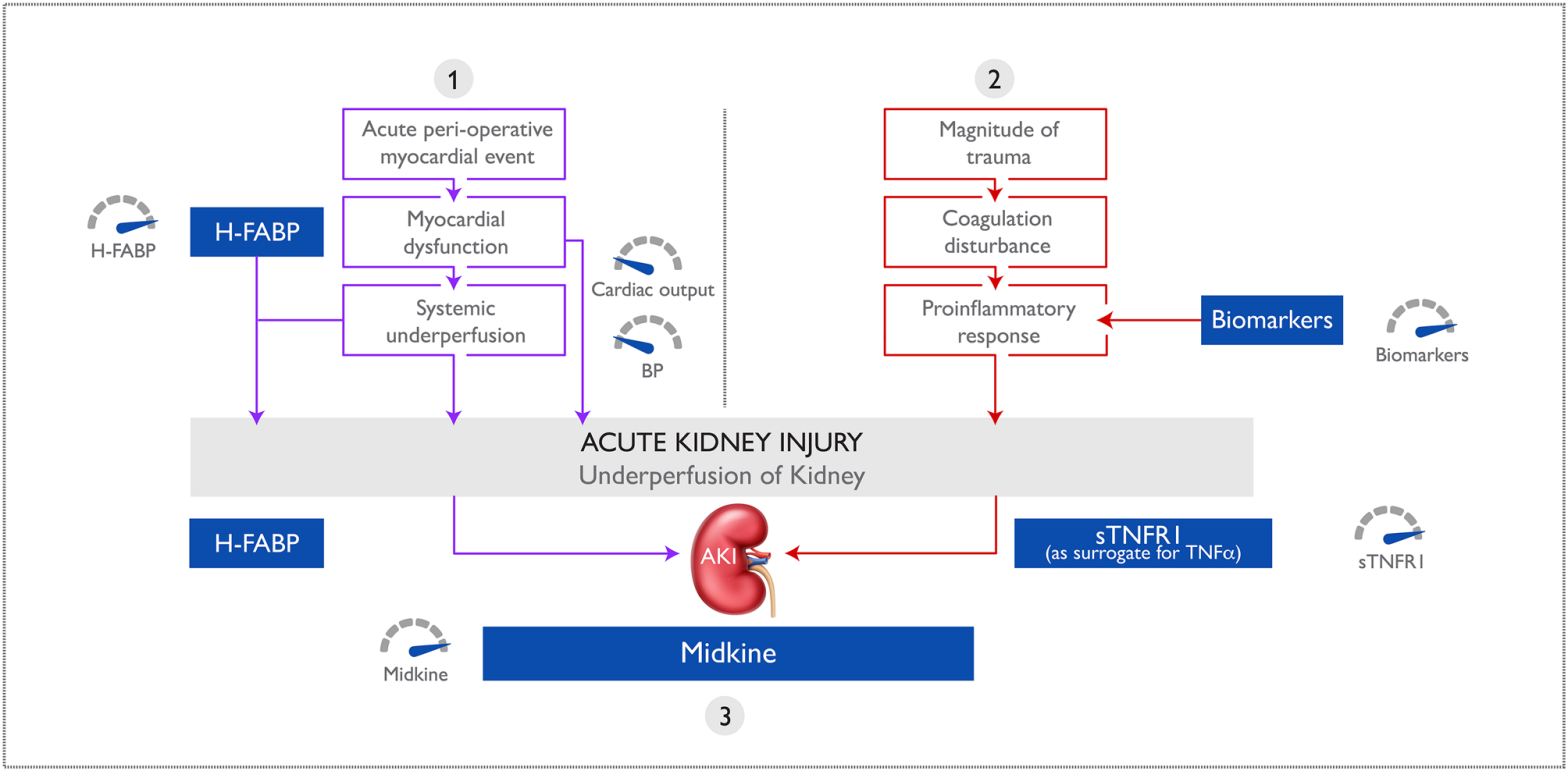
Pathogenesis of AKI. Three important pathways in the pathogenesis of AKI are represented by biomarkers in the model: (1) hypoperfusion (H-FABP), (2) proinflammation (sTNFR1 as a surrogate for the transient TNFα response) and (3) ischaemia reperfusion injury (MK). Adapted from McBride et al.^11^. *AKI* acute kidney injury, *BP* blood pressure, *H-FABP* heart-type fatty acid-binding protein, *MK* midkine, *sTNFR* soluble tumour necrosis factor receptor, *TNFα* tumour necrosis factor alpha.

Three important pathways involved in the pathogenesis of AKI were identified, namely hypoperfusion (H-FABP), ischaemia reperfusion injury (MK) and proinflammatory insult (sTNFR1) (Fig. 4).

Risk factors that have previously been reported for AKI include age, pre-existing CKD, male gender, diabetes, heart failure and surgery^15–17^. In this study, three clinical risk factors were identified for patients who were at potential risk for the development of AKI, pre and post operatively; age, dementia and hypertension (Table 4). Using biomarker data and clinical factors we developed a BRS and a CRS, respectively. Combining BRS (Table 3) with CRS (Table 4) grouped patients into four risk categories, each of which is associated with a clinical management regime (Table 5). Deployment of this proactive clinical AKI tool would allow clinicians to stratify patients at risk of AKI enabling early intervention and improving patient outcomes. Use of a cardiac proactive clinical AKI tool has been described previously^11^.

In the study cohort 63/201 (31.3%) orthopaedic trauma patients developed AKI post ORIF surgery. The incidence of AKI in this patient cohort is higher than previously reported^40–43^. Patients who developed AKI were significantly older and more likely to have hypertension and/or dementia. Advanced age is frequently reported as a risk factor for AKI^1^ however, to our knowledge, this is the first report that identified dementia as a potential risk factor in the development of AKI. Recently, an association of heightened proinflammatory activity in patients with dementia has been reported^44^. Our results are consistent with these findings. Interestingly, patients surviving AKI have a higher probability of developing dementia in the long-term compared to patients who did not develop AKI^45^.

The time between presentation and surgery was 2 days and was not significant between non AKI and AKI patients. An optimal operation time of between 24–48 h after orthopaedic trauma has been identified for lower extremity fracture fixation to reduce complications. Operations performed outside of this timeframe are associated with increased morbidity and mortality^46^.

AKI patients stayed an additional two days in hospital compared to non AKI patients (12.0 (3.7–20.3) days for AKI vs. 9.8 (1.9–17.7) days for non AKI patients), consistent with previous findings^47^. Patients that develop AKI following elective total joint arthroplasty also have increased hospital stay^48^. The management of patients with AKI is a significant burden to the healthcare service^49^. Earlier diagnosis and management of patients at risk of AKI will potentially reduce the financial burden on healthcare systems in addition to improving patient outcomes and welfare.

Surprisingly previous work has failed to identify hypotension as a serious risk factor in AKI^50^. Since intraoperative blood pressure modulation is a readily available strategy for anaestheologists, inability to show a link between hypotension and AKI at orthopaedic surgery could arguably generate a false sense of complacency. However, the reason for lack of the relationship between perioperative blood pressure and subsequent AKI could be because most patients have non-invasive blood pressure measurements where blood pressure readings are obtained by an arm cuff measurement every 5 min whereas more critically ill patients have continuous arterial blood pressure measurements which detect and record all hypotensive episodes. In Braüner’s study^50^ they recorded lowest blood pressure measurement intraoperatively. Their work suggested that this was not a useful marker in terms of AKI prediction. However, we argue that clinically significant hypotensive episodes may have been missed in this study if they happened in between measurements. This means that transient, albeit clinically significant, hypotension could be missed in between these times. In summary, the use of clinical data alone (including perioperative hypotensive events) to predict perioperative AKI is of limited usefulness in hip fracture surgery. It has already been shown in cardiac surgery that biomarkers of ischaemia reperfusion (MK) or hypotension (VEGF or HFABP) and inflammation augmented clinical parameters^11^. This present work suggests that this principle is also applicable to hip fracture surgery.

In a meta-analysis it was demonstrated that perioperative hemodynamic optimization in surgery patients, reduces post-operative acute renal injury^51^. Preoperative prediction would allow for enhanced perioperative hemodynamic optimization i.e. provision of Level 2 care provided post operatively, and invasive hemodynamic monitoring intraoperatively rather than blood pressure measurements every 5 min, as is routine for such cases in many centres. It could also be taken as a contraindication to non steroidal anti-inflammatory use post operation.

Biomarkers are not a substitute to the classical approach to using low-cost information—but add to the information available to the clinician. However, it must be noted that a clear clinical history in these elderly patients can sometimes be unreliable. Hence the need for the objective information that biomarkers provide.

### Limitations of the study

Clinical characteristics were not reliably available for everyone in this patient group including a guaranteed history of normal renal function pre trauma; patients were assumed, based on available clinical history, to have a normal renal function prior to their trauma and a baseline eGFR of at least 60 ml/min/1.73m^2^. Therefore, patients who had undiagnosed pre-trauma renal dysfunction could have been included in the study. Nevertheless, subsequent fluctuations in renal function were still detectable using our proactive clinical AKI tool, demonstrating the clinical utility of our proposed method in this patient cohort, where obtaining clinical history is sometimes challenging and unreliable.

## Conclusion

In conclusion, serum H-FABP and sTNFR1 measured pre operatively and serum H-FABP, MK and sTNFR1 measured post operatively, identified orthopaedic trauma patients at risk of developing AKI during ORIF surgery. Utilisation of the proactive clinical AKI tool, which combines BRS with CRS, would allow clinicians to stratify patients into one of four AKI risk categories with related treatment regimens that could impact patient care and management, length of hospital stay, and the efficient use of hospital resources.

## Supplementary information

Supplementary Information

## Data Availability

The datasets used and/or analysed during the current study are available from the corresponding author on reasonable request.

## References

[1] Haines RW, Fowler AJ, Kirwan CJ, Prowle JR (2019). The incidence and associations of acute kidney injury in trauma patients admitted to critical care: a systematic review and meta-analysis. J. Trauma Acute Care Surg..

[2] Bell S (2015). Risk of postoperative acute kidney injury in patients undergoing orthopaedic surgery–development and validation of a risk score and effect of acute kidney injury on survival: observational cohort study. BMJ.

[3] Grams ME (2016). Acute kidney injury after major surgery: a retrospective analysis of veterans health administration data. Am. J. Kidney Dis..

[4] Griffin, X. L., Parsons, N., Achten, J., Fernandez, M. & Costa, M. L. Recovery of health-related quality of life in a United Kingdom hip fracture population. *Bone Joint J.* **97**-**B**, 372–382 (2015). 10.1302/0301-620X.97B3.35738 25737522

[5] Kellum, J. A., Lameire, N. & KDIGO AKI Guideline Work Group. Diagnosis, evaluation, and management of acute kidney injury: a KDIGO summary (Part 1). *Crit. Care* **17**, 204 (2013). 10.1186/cc11454 PMC4057151 23394211

[6] Md Ralib A, Pickering JW, Shaw GM, Endre ZH (2013). The urine output definition of acute kidney injury is too liberal. Crit. Care.

[7] Parikh A (2017). Does NGAL reduce costs? A cost analysis of urine NGAL (uNGAL) and serum creatinine (sCr) for acute kidney injury (AKI) diagnosis. PLoS ONE.

[8] Bellomo R, Kellum JA, Ronco C (2004). Defining acute renal failure: physiological principles. Intensive Care Med..

[9] Bennett M (2008). Urine NGAL predicts severity of acute kidney injury after cardiac surgery: a prospective study. Clin. J. Am. Soc. Nephrol..

[10] Liangos O (2009). Comparative analysis of urinary biomarkers for early detection of acute kidney injury following cardiopulmonary bypass. Biomarkers.

[11] McBride WT (2019). Stratifying risk of acute kidney injury in pre and post cardiac surgery patients using a novel biomarker-based algorithm and clinical risk score. Sci. Rep..

[12] McBride WT (2013). Cytokine phenotype, genotype, and renal outcomes at cardiac surgery. Cytokine.

[13] Mishra J (2005). Neutrophil gelatinase-associated lipocalin (NGAL) as a biomarker for acute renal injury after cardiac surgery. Lancet.

[14] Parikh CR (2011). Postoperative biomarkers predict acute kidney injury and poor outcomes after adult cardiac surgery. J. Am. Soc. Nephrol..

[15] Overview Acute kidney injury: prevention, detection and management NICE Guideline https://www.nice.org.uk/guidance/ng148#:~:text=Acute%20kidney%20injury%3A%20prevention%2C%20detection%20and%20management,NICE%20guideline%20%5BNG148&text=It%20aims%20to%20improve%20assessment,people%20with%20acute%20kidney%20injury (2019).

[16] Kheterpal S (2007). Predictors of postoperative acute renal failure after noncardiac surgery in patients with previously normal renal function. Anesthesiology.

[17] Kheterpal S (2009). Development and validation of an acute kidney injury risk index for patients undergoing general surgery. Anesthesiology.

[18] Abdulla A (2017). Proceedings from the symposium on kidney disease in older people: Royal Society of Medicine, London, January 19, 2017. Gerontol. Geriatr. Med..

[19] Myers GL (2006). Recommendations for improving serum creatinine measurement: a report from the laboratory working group of the national kidney disease education program. Clin. Chem..

[20] White SM, Rashid N, Chakladar A (2009). An analysis of renal dysfunction in 1511 patients with fractured neck of femur: the implications for peri-operative analgesia. Anaesthesia.

[21] Lopes JA, Jorge S (2013). The RIFLE and AKIN classifications for acute kidney injury: a critical and comprehensive review. Clin. Kidney J..

[22] R Core Team. R: A Language and Environment for Statistical Computing. (2018).

[23] Barr ELM (2018). High baseline levels of tumor necrosis factor receptor 1 are associated with progression of kidney disease in indigenous Australians with diabetes: the eGFR follow-up study. Diabetes Care.

[24] Carlsson, A. C. *et al.* Association of soluble tumor necrosis factor receptors 1 and 2 with nephropathy, cardiovascular events, and total mortality in type 2 diabetes. *Cardiovasc. Diabetol.* **15**, (2016). 10.1186/s12933-016-0359-8 PMC4770690 26928194

[25] Carlsson AC (2015). Soluble tumor necrosis factor receptor 1 is associated with glomerular filtration rate progression and incidence of chronic kidney disease in two community-based cohorts of elderly individuals. Cardiorenal Med..

[26] Bradley JR, Thiru S, Pober JS (1995). Disparate localization of 55-kd and 75-kd tumor necrosis factor receptors in human endothelial cells. Am. J. Pathol..

[27] Al-Lamki RS, Mayadas TN (2015). TNF receptors: signaling pathways and contribution to renal dysfunction. Kidney Int..

[28] Gohda T (2012). Circulating TNF receptors 1 and 2 predict stage 3 CKD in type 1 diabetes. J. Am. Soc. Nephrol..

[29] Miyazawa I (2011). Association between serum soluble TNFα receptors and renal dysfunction in type 2 diabetic patients without proteinuria. Diabetes Res. Clin. Pract..

[30] Wu X, Guo R, Chen P, Wang Q, Cunningham PN (2009). TNF induces caspase-dependent inflammation in renal endothelial cells through a Rho- and myosin light chain kinase-dependent mechanism. Am. J. Physiol. Renal Physiol..

[31] Baker RC (2006). Methylprednisolone increases urinary nitrate concentrations and reduces subclinical renal injury during infrarenal aortic ischemia reperfusion. Ann. Surg..

[32] Muehlschlegel JD (2010). Heart-type fatty acid binding protein is an independent predictor of death and ventricular dysfunction after coronary artery bypass graft surgery. Anesth. Analg..

[33] Oezkur M (2014). Preoperative serum h-FABP concentration is associated with postoperative incidence of acute kidney injury in patients undergoing cardiac surgery. BMC Cardiovasc. Disord..

[34] Kamijo-Ikemori A, Sugaya T, Kimura K (2006). Urinary fatty acid binding protein in renal disease. Clin. Chim. Acta.

[35] Pelsers MMAL, Hermens WT, Glatz JFC (2005). Fatty acid-binding proteins as plasma markers of tissue injury. Clin. Chim. Acta.

[36] Veerkamp JH (1990). Detection, tissue distribution and (sub)cellular localization of fatty acid-binding protein types. Mol. Cell. Biochem..

[37] Andreucci M, Faga T, Pisani A, Perticone M, Michael A (2017). The ischemic/nephrotoxic acute kidney injury and the use of renal biomarkers in clinical practice. Eur. J. Intern. Med..

[38] Sato W, Sato Y (2014). Midkine in nephrogenesis, hypertension and kidney diseases. Br. J. Pharmacol..

[39] Haffner-Luntzer M (2014). Midkine-deficiency delays chondrogenesis during the early phase of fracture healing in mice. PLoS ONE.

[40] Bennet SJ, Berry OMB, Goddard J, Keating JF (2010). Acute renal dysfunction following hip fracture. Injury.

[41] Ersoy A (2003). Survival analysis of the factors affecting in mortality in injured patients requiring dialysis due to acute renal failure during the Marmara earthquake: survivors vs non-survivors. Clin. Nephrol..

[42] Kimmel LA, Wilson S, Janardan JD, Liew SM, Walker RG (2014). Incidence of acute kidney injury following total joint arthroplasty: a retrospective review by RIFLE criteria. Clin. Kidney J..

[43] Sykes L, Kalra PA, Green D (2019). Comparison of impact on death and critical care admission of acute kidney injury between common medical and surgical diagnoses. PLoS ONE.

[44] Kim J-W (2018). Longitudinal associations between serum cytokine levels and dementia. Front. Psychiatry.

[45] Tsai H-H, Yen R-F, Lin C-L, Kao C-H (2017). Increased risk of dementia in patients hospitalized with acute kidney injury: a nationwide population-based cohort study. PLoS ONE.

[46] Sangkomkamhang, T., Thinkhamrop, W., Thinkhamrop, B. & Laohasiriwong, W. Incidence and risk factors for complications after definitive skeletal fixation of lower extremity in multiple injury patients: a retrospective chart review. *F1000Research* **7**, 612 (2018). 10.12688/f1000research.14825.1 PMC5981188 29904601

[47] Porter CJ (2017). Acute and chronic kidney disease in elderly patients with hip fracture: Prevalence, risk factors and outcome with development and validation of a risk prediction model for acute kidney injury. BMC Nephrol..

[48] Abar O, Toossi N, Johanson N (2018). Cost and determinants of acute kidney injury after elective primary total joint arthroplasty. Arthroplast. Today.

[49] College of Physicians, R. *Falls and fragility fracture audit programme national hip fracture database (NHFD) annual report 2017*. (2017).

[50] Braüner Christensen J (2020). Predictors of acute kidney injury after hip fracture in older adults. Geriatr. Orthop. Surg. Rehabil..

[51] Brienza N, Giglio MT, Marucci M, Fiore T (2009). Does perioperative hemodynamic optimization protect renal function in surgical patients? A meta-analytic study. Crit. Care Med..

