# Supplementary material for: Acute kidney injury risk in orthopaedic trauma patients pre and post surgery using a biomarker algorithm and clinical risk score
Source: Sci Rep. 2020 Nov 17;10:20005. doi: 10.1038/s41598-020-76929-y (PMC7673130; doi:10.1038/s41598-020-76929-y)
Supplement: Supplementary file 1 — Supplementary Information [file 41598_2020_76929_MOESM1_ESM.pdf]

Supplementary information

AKI risk in orthopaedic trauma patients pre and post surgery using a biomarker algorithm and clinical risk score

Mary Jo Kurth[1], William T. McBride[2], Gavin McLean[3], Joanne Watt[1], Anna Domanska[1], John V. Lamont[1], Daniel Maguire[1], Peter Fitzgerald[1], and Mark W. Ruddock[1]*.

Supplementary Note 1. Worked Examples

Post operative worked examples BRS and CRS for patient F036 (non AKI) and BRS and CRS for patient F079 (AKI)

Two patients, one non AKI and one with AKI, were randomly chosen from the database. The data from each patient post trauma was applied to the BRS and CRS to demonstrate the clinical utility of the AKI tool in routine practice.

For the non AKI patient the CRS was low and post operatively the BRS was negative (-5.50), therefore, the patient would be placed in category 1 “routine management”. A category 1 assignment places the patient in the lowest risk category for the development of AKI and routine management is sufficient.

For the AKI patient, the CRS was high; age and hypertension (2/3 risk factors) and the BRS was positive (3.60). This patient was assigned to category 4 “highest risk management”. A category 4 assignment informs the clinician that this patient should be closely monitored for AKI and early intervention/dialysis may be necessary.

Supplementary Note 2. Worked example BRS and CRS for patient F036 (non AKI)

Supplementary Table S1. BRS Post operative

Biomarker	Result	Patient Score* Cut-off (BRS)
H-FABP	6.47	
sTNFR1	0.55	
MK	211.43	
Patient Score*	-5.5	**< -1.05 (Negative)**

*Patient Score = -8.185 + 2.037*ln(H-FABP) + 2.373*ln(sTNFR1) + 0.056*ln(MK)

Supplementary Table S2. CRS Post operative

Clinical Factor	Level	CRS
Age = 49.0	**<80**<br>≥80	**0**<br>1
Dementia = 0	**No**<br>Yes	**0**<br>1
Hypertension = 0	**No**<br>Yes	**0**<br>1
	**Total**	**0 (Low)**

Supplementary Table S3. Post operative BRS and CRS:

Category	BRS	CRS	Clinical Management
**1**	**Negative**	**Low**	**Routine management**
2	Negative	High	Assign to low risk management
3	Positive	Low	Assign to higher risk management
4	Positive	High	Assign to highest risk management

Supplementary Note 3. Worked example BRS and CRS for patient F079 (AKI)

Supplementary Table S4. BRS Post operative

Biomarker	Result	Patient Score* Cut-off (BRS)
H-FABP	136.58	
sTNFR1	1.72	
MK	5297.70	
Patient Score*	3.60	**≥ -1.05 (Positive)**

*Patient Score = -8.185 + 2.037*ln(H-FABP) + 2.373*ln(sTNFR1) + 0.056*ln(MK)

Supplementary Table S5. CRS Post operative

Clinical Factor	Level	CRS
Age = 87.6	<80<br>**≥80**	0<br>**1**
Dementia = 0	**No**<br>Yes	**0**<br>1
Hypertension = 1	No<br>**Yes**	0<br>**1**
	**Total**	**2 (High)**

Supplementary Table S6. Post operative BRS and CRS:

Category	BRS	CRS	Clinical Management
1	Negative	Low	Routine management
2	Negative	High	Assign to low risk management
3	Positive	Low	Assign to higher risk management
**4**	**Positive**	**High**	**Assign to highest risk management**

Supplementary Note 4. Distribution of AKI and non AKI patients within risk categories

The risk of developing AKI was low for patients who were categorised either 1 or 2. Whereas, patients categorised 3 or 4 were at higher risk for developing AKI. If the BRS was negative, patients were categorised either 1 or 2. A combination of BRS and CRS categorised >95% of non AKI patients in category 1 and >87% in category 2. Thus, a combination of BRS and CRS identified patients that were at lower risk of developing AKI and assigned them to the low risk clinical management category. Patients with positive BRS were assigned to either category 3 or 4, high risk.

Supplementary Table S7. Distribution of AKI and non AKI patients within risk categories for AKI 1-4

	**Category 1**	**Category 2**	**Category 3**	**Category 4**	**Total**
**Non AKI**	21 (95.5%)	41 (87.2%)	5 (83.3%)	19 (36.5%)	86 (78.6%)
**AKI**	1 (4.5%)	6 (12.8%)	1 (16.7%)	33 (63.5%)	41 (21.4%)
**Total**	22 (100%)	47 (100%)	6 (100%)	52 (100%)	127 (100%)

Data refers to number and percentage of patients within each risk category identified as AKI or non AKI using a combination of postoperative BRS (H-FABP + sTNFR1 + MK) and CRS.

AKI = acute kidney injury, H-FABP = heart-type fatty acid-binding protein, sTNFR = soluble tumour necrosis factor receptor, MK = midkine, BRS = biomarker risk score, CRS = clinical risk score
